# Comparative genomics of hypervirulent *Klebsiella pneumoniae* isolates from Brazil

**DOI:** 10.1007/s00203-026-05262-1

**Published:** 2026-10-12

**Authors:** Felipe G. Sacoda, João V. O. Cruz, Itana A. Santos, Adriano S. S. Monteiro, Amanda O. S. Melo, Danilo J. P. G. Rocha, Emilia M. M. A. Belitardo, Flavia F. Aburjaile, Siomar C. Soares, Mateus M. Costa, Aristóteles Góes-Neto, Rommel T. J. Ramos, Bertram Brenig, Vasco Azevedo, Joice N. Reis, Luis G. C. Pacheco

**Affiliations:** 1 Federal University of Bahia, Salvador, BA Brazil; 2 Gonçalo Moniz Institute, Oswaldo Cruz Foundation (FIOCRUZ), Salvador, BA Brazil; 3 Faculty of Pharmacy, Federal University of Bahia, Salvador, BA Brazil; 4 Institute of Health Sciences, Federal University of Bahia, Salvador, BA Brazil; 5 Department of Preventive Veterinary Medicine, Federal University of Minas Gerais, Belo Horizonte, MG Brazil; 6 Institute of Biological and Natural Sciences, Federal University of Triângulo Mineiro, Uberaba, MG Brazil; 7 Federal University of the São Francisco Valley, Petrolina, PE Brazil; 8 Institute of Biological Sciences, Federal University of Minas Gerais, Belo Horizonte, MG Brazil; 9 Federal University of Pará, Belém, PA Brazil; 10 Institute of Veterinary Medicine, University of Göttingen, 37073 Göttingen, Germany

**Keywords:** *Klebsiella pneumoniae*, Hypervirulent, Comparative genomics, Pangenome

## Abstract

*Klebsiella pneumoniae* represents a major public health threat at the convergence of antimicrobial resistance and hypervirulence. While classical *K. pneumoniae* (cKp) is a leading cause of healthcare-associated infections, hypervirulent *K. pneumoniae* (hvKp) causes severe invasive disease in otherwise healthy individuals. We performed a comparative genomic analysis of hvKp (*n* = 8) and cKp (*n* = 17) isolates recovered in Salvador, Brazil. The eight Brazilian hvKp isolates displayed the canonical hypervirulence genotype, encoding the five major biomarkers (*iroB*,* iucA*, *peg-344*, *rmpA*, and *rmpA2*) while carrying only a limited repertoire of intrinsic antimicrobial resistance genes. These isolates belonged to sequence types ST23, ST65, ST86, ST3690, and ST5750 and were associated with K1 or K2 capsular types and the O1 antigen. In three isolates, *iroB* was detected in both chromosomal and plasmid-associated regions, a dual localization reported at low frequency among hvKp. The predominant plasmid replicons were IncHI1B (pNDM-MAR) and repB_KLEB, with IncFIB (K) in two isolates. Screening for mobile genetic elements recovered an F-type conjugative transfer locus in these two isolates only. Chromosomes and plasmids showed high similarity with well-characterized reference hvKp strains, including SGH10 and NTUH-K2044, as well as the pLVPK virulence plasmid. Comparative proteomics analysis identified 53 protein clusters conserved across hvKp strains examined. Among these were four hypothetical proteins previously associated with invasive liver infection, containing DUF1471, DUF417, and carboxymuconolactone decarboxylase-related domains, suggesting potential roles in pathogenicity. Collectively, these findings expand current knowledge of hvKp genetic diversity in Salvador, Brazil and highlight genomic features that may contribute to the emergence and maintenance of the hypervirulent phenotype.

**Supplementary Information:**

The online version contains supplementary material available at https://doi.org/10.1007/s00203-026-05262-1.

## Introduction

*Klebsiella pneumoniae* is a critical priority pathogen at the intersection of antimicrobial resistance (AMR) and hypervirulence. Classical *K. pneumoniae* (cKp) is a major cause of healthcare-associated infections, whereas hypervirulent *K. pneumoniae* (hvKp) can cause invasive community-acquired infections, even in otherwise healthy individuals (Russo and Marr 2019; Stanton and Wyres 2024; Monteiro et al. 2024, 2025). Hypervirulent lineages are typically characterized by enhanced capsule production, hypermucoviscosity, and highly efficient iron-acquisition systems (Russo et al. 2024b).

Although hvKp was first reported in East Asia, convergent clones combining multidrug resistance and hypervirulence (MDR-hvKp) have now been reported worldwide (Dong et al. 2022; Li et al. 2025; Raro et al. 2023). These convergence events pose an escalating public health threat, particularly with the emergence and international dissemination of carbapenem-resistant hvKp (CR-hvKp) strains (Chen et al. 2024; Maguire et al. 2025; Raro et al. 2023).

Since the first description of the genomic features associated with hypervirulence (Brisse et al. 2009), substantial progress has been made in defining the genetic determinants that characterize hvKp. More recently, Russo and colleagues (Russo et al. 2024a), proposed a standardized genomic definition based on five key biomarkers (*iroB*, *iucA*, *peg-344*, *rmpA*, and *rmpA2*), and demonstrated their functional relevance using mutant models.

Despite these advances, important knowledge gaps remain regarding the molecular basis of hypervirulence (Stanton and Wyres 2024). The presence of virulence plasmid-associated biomarkers alone does not fully explain the hypervirulent phenotype, suggesting that additional chromosomal and plasmid-encoded factors may contribute to pathogenicity. Furthermore, distinct virulence plasmid lineages may harbor different repertoires of virulence-associated genes, highlighting the complexity of hvKp evolution and adaptation (Stanton and Wyres 2024).

In this study, we performed comparative genomics and pangenome analyses on eight hypervirulent *Klebsiella pneumoniae* (hvKp) and 17 classical *K. pneumoniae* (cKp) isolates recovered in Salvador, Brazil, and compared them with 16 publicly available hvKp genomes. Our objectives were to characterize the genetic diversity of local hvKp lineages, besides identifying their resistance and virulence determinants. Additionally, we discuss aspects that remain poorly resolved, including the putative function of hvKp-enriched hypothetical proteins that lie outside the biomarker set established by Russo et al. (2024a), and the genomic localization of the *iro* locus in isolates carrying both chromosomal and plasmid-borne copies.

## Materials and methods

### Bacterial whole-genome sequencing and retrieval of public genomic data from hvKp isolates

A total of 41 *K. pneumoniae* genomes were included in the comparative genomic analyses. These comprised 25 isolates obtained in Salvador, Brazil, of which eight exhibited a hypervirulent phenotype based on a positive string test and the presence of the biomarkers *iucA*, *iroB*, *peg-344*, *rmpA*, and *rmpA2*. Five of these eight isolates were recovered from invasive meningitis infections, whereas the remaining three were classified as hvKp only after whole-genome sequencing (WGS) analysis. Furthermore, we included 16 publicly available genomes from hvKp isolates retrieved from Russo et al. (2024a), which were selected because they had undergone phenotypic testing confirming the hypervirulent phenotype (BioProject PRJNA981468). All isolates from Salvador, Brazil were identified and assessed by antimicrobial susceptibility testing (AST) using the Vitek^®^ 2 system (BioMérieux^™^), and their genomic DNA were extracted with the QIAamp DNA Mini Kit (Qiagen). Whole-genome sequencing libraries were prepared following the standard Illumina HiSeq paired-end 2 × 150 bp protocol, yielding read coverages greater than 300× per genome. Fifteen of the Brazilian genomes were obtained from a previous study by our research group under Bioproject PRJEB90215.

### Genome assembly, annotation, and detection of resistance and virulence genes

Raw sequencing reads were processed using the BV-BRC Comprehensive Genome Analysis pipeline with default parameters (Olson et al. 2023), which incorporates TrimGalore v0.6.5, Unicycler v0.4.8, Pilon v1.23, QUAST v5.2.0, and CheckM2 v1.1. Genome annotations were performed using the RAST toolkit. Antimicrobial-resistance genes (ARGs) were predicted using ResFinder v4.2.2 (Bortolaia et al. 2020; RRID: SCR_024314) and AbritAMR v1.0.15 (Sherry et al. 2023). Virulence-associated genes were screened using PathogenWatch v23.4.4, Kleborate v3.2.4 (RRID: SCR_024051), and Kaptive v3.1.0 (RRID: SCR_024046) (Wyres et al. 2016), focusing on canonical hvKp markers *iucA*,* iroB*,* rmpA/rmpA2*. The presence of additional hypervirulence-associated genes including *iroD* and *peg-344*, was confirmed by tBLASTx analysis using a minimum nucleotide identity threshold of 95%.

### Genotyping and plasmid profiling

Sequence types (ST), capsular (K) and lipopolysaccharide (O) loci, and plasmid replicons profiles were determined using PathogenWatch, which incorporates Kleborate v3.2.4 for genomic characterization. Plasmid and chromosomal -derived contigs were classified using mlplasmids v2.1.0 (Arredondo-Alonso et al. 2018). Mobile genetic elements and plasmid transfer functions were predicted in the eight Brazilian hvKp genomes. Contigs classified as plasmid-derived were annotated against mobileOG-db (Brown et al. 2022) through the Proksee platform (Grant et al. 2023). Conjugative and mobilizable systems were predicted using the CONJScan module (model set v2.1.0) implemented in MacSyFinder v2.1.6 (Néron et al. 2023; Cury et al. 2020), run in ordered-replicon mode against the CONJScan/Plasmids models.

### Pangenome analysis and functional annotation

Protein-coding sequences from all genomes were clustered using BPGA v1.3.0 (Chaudhari et al. 2016) and OrthoVenn3 (Sun et al. 2023) to define core, accessory, and unique genes sets. Functional annotation was performed with EggNOG-mapper v2.1.12 (Cantalapiedra et al. 2021), and genes were assigned to functional categories based on the Clusters of Orthologous Groups (COG) database. The distribution of COG categories was subsequently compared across core, accessory, and unique genome fractions.

### Comparative genomics of siderophore *loci*

The genomic organization of the salmochelin-associated *iroB* and *iroD* genes was investigated in Brazilian hvKp isolates. Genomic regions harboring these *loci* were reconstructed and compared with those of the reference hvKp strains NTUH-K2044 and SGH10 and the virulence plasmid pLVPK. Syntenic alignments were generated using CAGECAT (Gilchrist et al. 2021) to assess gene conservation and structural variation within the *iro* locus. Circular genome and plasmid maps were produced using Proksee (Grant et al. 2023).

### Functional annotation of hypothetical proteins

The hypothetical proteins from hvKp strains described in the study by Spadar et al. (2022) were further characterized following the PROST protocol (Kilinc et al. 2022) integrating sequence-based homology search by BLASTp, and structure-based search using Foldseek (Van Kempen et al. 2024), to identify the most likely candidate homologs. For each candidate homolog, three-dimensional protein structures were predicted using AlphaFold 3 (Abramson et al. 2024). Predicted structures were then aligned with their corresponding candidate homologs using FATCAT, and the resulting p-values were used to assess confidence in the structural alignment (Li et al. 2020). The homolog with the lowest FATCAT p-value across the evaluated prediction sources was selected as the best match. Structural superpositions were visualized using Mol* Viewer (Sehnal et al. 2021).

## Results and discussion

### The pangenome structure of hvKp isolates

All 25 *K. pneumoniae* isolates from Brazil generated high-quality genome assemblies, with N50 values ranging from 170 to 499 kb; GC contents ~ 57.0%; and completeness ≥ 99.0%. Genome sizes ranged from 5.3 to 5.9 Mb with ~ 5,200–6,000 CDS, consistent with previously reported genomes of the *K. pneumoniae* species complex (Wyres et al. 2020) (Supplementary Table S1).

**Fig. 1:**
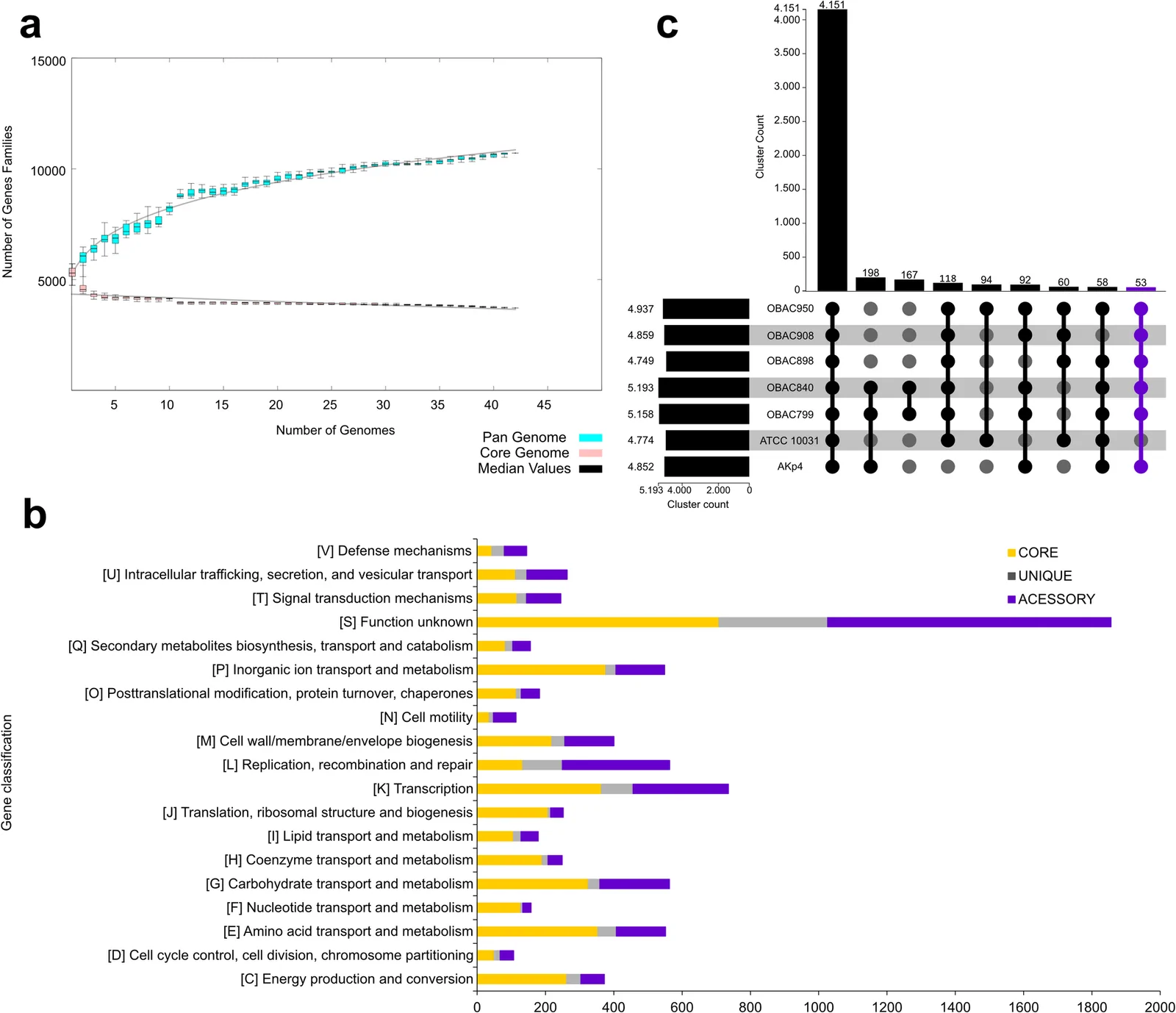
Pangenome, functional annotation and protein clustering of *Klebsiella pneumoniae* isolates. (**A**) Gene families as a function of the number of genomes in the *K. pneumoniae* pangenome, including classical and hypervirulent strains (*n* = 41); (**B**) Upset plot of protein clusters from hypervirulent *K. pneumoniae* strains and the ATCC 10,031 reference. The dataset includes the control strain (ATCC 10031), one hypervirulent strain from the literature (AKp4), and isolates originating from Brazil; (**C**) Distribution of Clusters of Orthologous Genes (COGs) among the essential, accessory, and unique genes of the *K. pneumoniae* pangenome

The aggregated dataset (*n* = 41), which included publicly available hvKp genomes, identified 10,686 gene families. Of these 3,720 (34.8%) constituted the core genome, supporting the well-established open pangenome nature of *K. pneumoniae* (Fig. 1A). In addition, 881 genes families were exclusively shared among the hvKp isolates included in this study, suggesting the presence of lineage-specific genetic traits potentially associated with hypervirulence, ecological adaptation, and host interaction.

Functional classification of the pangenome (Fig. 1B) revealed an overrepresentation of genes associated with replication, recombination, and repair (COG category L), transcription (K), and amino-acid transport and metabolism (E) within the unique genome fraction. This pattern is consistent with the remarkable genomic plasticity of *K. pneumoniae* and its capacity to acquire genetic material through horizontal gene transfer (HGT), as previously reported (Russo and Marr 2019; Navon-Venezia et al. 2017). Notably, nearly 30% of accessory genes were assigned to the” function unknown” category, highlighting the substantial proportion of uncharacterized genetic content within *K. pneumoniae* pangenome. These genes may encode previously unrecognized virulence-associated functions, regulatory mechanisms, or mobile genetic elements that contribute to adaptation and pathogenicity.

### Genomic features of hypervirulent isolates

Eight Brazilian isolates, five pre-selected based on infection site and three through genomic screening, fulfilled both the phenotypic (positive string test) and genomic criteria for hvKp, harboring the canonical virulence determinants *iucA*,* iroB*,* rmpA/A2*, and *peg-344* (Table 1). In contrast, these isolates carried only a limited repertoire of antimicrobial resistance genes, primarily the intrinsic determinants *bla*_*SHV*_, *fosA*,* oqxAB*, and e*mrD* (Ito et al. 2017; Li et al. 2023). The presence of intrinsic *bla*_*SHV*_ in the absence of extended-spectrum β-lactamase (ESBL) or carbapenemase genes supports their antimicrobial-susceptible yet highly virulent phenotype.

**Table 1:** Virulence biomarkers, sequence type (ST), capsular (K) and lipopolysaccharide (O) loci, and plasmid replicon profile of the eight sequenced hypervirulent *K. pneumoniae* (hvKp) isolates from this study

Sample	Sequence type	Aerobactin	AbST	Salmochelin	SmST	peg-344	rmpADC/rmpA2^a^	O-type	Capsule type	Inc Match
OBAC799	23	*iuc 1*	1	*iro 1*	2	Present	rmp 1; KpVP-1/*rmpA2*_6*/60%	O1;O2v2/O1	KL1/K1	IncHI1B(pNDM-MAR)1, repB_KLEB
OBAC840	23	*iuc 1*	1	*iro 1*	2	Present	rmp 1; KpVP-1/*rmpA2*_6*/60%	O1;O2v2/O1	KL1/K1	IncHI1B(pNDM-MAR)1, repB_KLEB, IncFIB(pKPHS1)1
OBAC898	3690	*iuc 2*	14-2LV	*iro 2*	27	Present	rmp 2; KpVP-2/*-*	O1;O2v1/O1	KL2/K2	IncFIB(K)_1
OBAC908	65	*iuc 1*	1	*iro 1*	1	Present	rmp 1; KpVP-1 (truncated)/*rmpA2*_6*/47%	O1;O2v2/O1	KL2/K2	repB_KLEB, IncHI1B(pNDM-MAR)1
OBAC950	86	*iuc 1*	1	*iro 1*	1	Present	rmp 1; KpVP-1/*rmpA2*_9*/54%	O1;O2v1/O1	KL2/K2	repB_KLEB, IncHI1B(pNDM-MAR)1
HB103	5750	*iuc 2*	3-2LV	*iro 2*	4-2LV	Present	*rmp* 2; KpVP-2/-	O1;O2v2/O1	KL2/K2	IncFIB(K)1
ITUAC436	23	*iuc 1*	1	*iro 2*	2	Present	*rmp* 1; KpVP-1/rmpA2_6/60%	O1;O2v2/O1	KL1/K1	repB_KLEB_VIR
ITUAC449	65	*iuc 1*	1	*iro 2*	1	Present	*rmp* 1; KpVP-1 (truncated)/-	O1;O2v2/O1	KL2/K2	IncHI1B(pNDM-MAR)1, repB_KLEB

^a^Percentage of sequence length covered, measured from the start codon of *rmpA2* gene

Detected sequence types were ST23, ST65, ST86, ST3690, and ST5750 across the eight sequenced hvKp isolates, the first three of which are frequently associated with hvKp lineages (Lee et al. 2017; Russo et al. 2024b). Furthermore, ST3690 is a single-locus variant of ST380, differing at only one of the seven housekeeping genes used in the MLST scheme. ST380 belongs to CG380, a clonal group associated with the K2 capsular type (Rodríguez-Medina et al. 2024; Medina et al. 2025). Although CG380 is less frequently implicated than CG23 and CG65 (Wyres et al. 2020), hypervirulent isolates of this clonal group have been reported in Europe, in France (Hentzien et al. 2017) and Portugal (Loiodice et al. 2025), and in Latin America, in Mexico (Rodríguez-Medina et al. 2024) and Colombia (Medina et al. 2025). Clinical and epidemiological data specific to ST3690 nevertheless remain scarce. Capsule *loci* K1 and K2, together with the O1 antigen locus, predominated among the isolates, further corroborating hypervirulent identity (Table 1). These genomic features are consistent with what has been described for hvKp lineages elsewhere, in which ST23-K1 and ST65-K2 lineages remain the most prevalent and clinically relevant hypervirulent clones (Dong et al. 2022; Wyres et al. 2020).

Interestingly, *iroB* was identified in both chromosomal and plasmid-associated genomic regions in OBAC799, OBAC840 and ITUAC436. This distribution may reflect recent gene duplication events, recombination, or mobilization mediated by mobile genetic elements. The co-occurrence of chromosomal and plasmid-borne *iro loci* has been reported previously in hvKp, although it appears to occur at a lower frequency (Lam et al. 2018a, b).

### Plasmid replicons and conjugative modules

Plasmid replicon analysis identified the widespread presence of IncHI1B(pNDM_MAR)-like and repB_KLEB replicons among the Brazilian hvKp isolates. repB_KLEB-type plasmid backbones are commonly associated with the carriage of virulence determinants, whereas pNDM_MAR-like plasmids have been implicated in the acquisition of both virulence and antimicrobial resistance genes (Navon-Venezia et al. 2017; Arcari and Carattoli 2023; Spadar et al. 2023; Zhang et al. 2024). Plasmids carrying these replicons are epidemiologically relevant: an EURGen-Net investigation reported backbones of this type carrying the aerobactin operon together with *bla*_OXA−48_ across multiple European countries (Linkevicius et al. 2025). Zhang and colleagues further characterized an IncHI1B(pNDM_MAR)/repB hybrid plasmid co-harboring *bla*_KPC−2_ and virulence determinants, illustrating that backbones of this type can accommodate both resistance and virulence genes (Zhang et al. 2024).

No conjugative modules were detected in the isolates carrying IncHI1B(pNDM_MAR)/repB_KLEB-type replicons, consistent with the general observation that pLVPK-like virulence plasmids lack a complete *tra* cluster and are not self-transmissible. In contrast, sequences encoding type IV secretion system components were detected in the isolates OBAC898 and HB103, both carrying IncFIB(K) replicons together with the canonical hvKp biomarkers. The presence of these modules does not establish transfer capacity, but isolates carrying comparable plasmid backbones are of concern in healthcare settings where MDR *K. pneumoniae* circulate, since non-self-transmissible virulence plasmids of this type have been shown to transfer with the assistance of a helper plasmid (Zhang et al. 2024). This finding underscores the importance of continuous genomic surveillance to detect early convergence between hypervirulence and antimicrobial resistance and to monitor the emergence of MDR-hvKp lineages in Brazil.

### Comparative proteomics and unique hvKp protein clusters

Ortholog clustering analysis performed with OrthoVenn3 identified 4,151 protein clusters shared across all analyzed *K. pneumoniae* genomes, representing the conserved proteomic core of the dataset. In addition, 53 protein clusters were exclusively detected in hvKp isolates (Fig. 1C). Importantly, approximately 68% of these hvKp-specific proteins were plasmid-encoded, underlining the pivotal role of Kp virulence plasmids (KpVP) in shaping the hypervirulent phenotype (Russo et al. 2024a; Li et al. 2025).

**Fig. 2:**
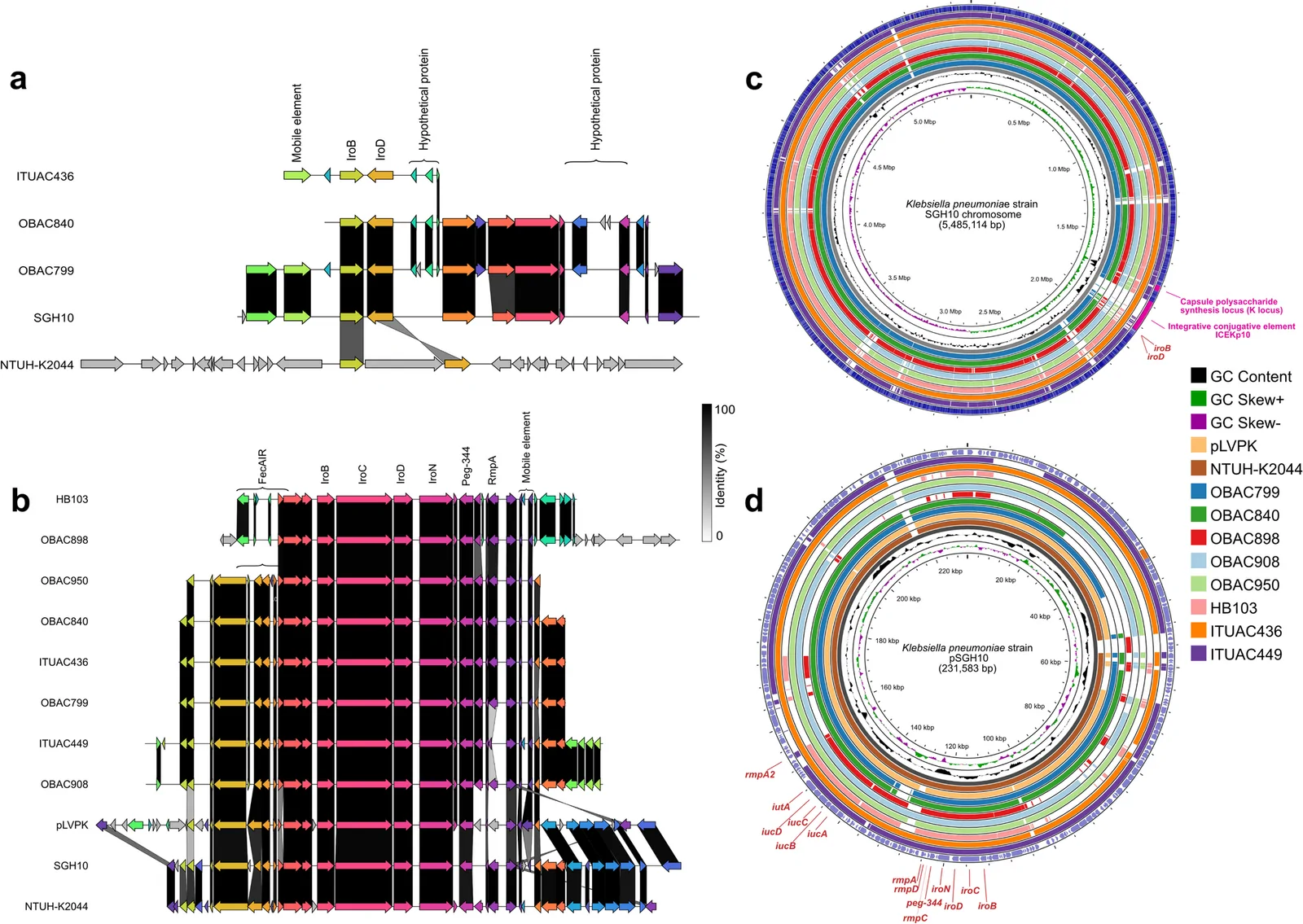
Synteny and circular genome visualization of hypervirulent *Klebsiella pneumoniae* isolates. (**A**) Genomic context of the *iroB* and *iroD* genes located in the chromosome and (**B**) plasmid in sequenced hypervirulent *K. pneumoniae* isolates, SGH-10, NTUH-K2044 and pLVPK. (**C**) Circular genome representation of chromosome-associated and (**D**) plasmid-associated sequences

Among the 53 hvKp-associated protein clusters (Supplementary Table S2), the salmochelin biosynthesis markers *iroB* and *iroD* were identified. These genes were detected on both chromosomal and plasmid-derived sequences. In three isolates (OBAC799, OBAC840, and ITUAC436), *iroB* and *iroD* were present at both genomic locations simultaneously, indicating the coexistence of chromosomal and plasmid-borne copies of the *iro* locus (Fig. 2A, B).

While the presence of multiple copies of antimicrobial resistance genes (ARGs) is well known to confer increased resistance to specific antibiotic classes, a comparable effect has not been well characterized for virulence genes, particularly when chromosomal and plasmid-borne copies coexist in the same isolate. Nevertheless, the presence of multiple copies of *iroB* and *iroD*, one chromosomal and one plasmid-borne, may increase gene dosage, with a potential effect on the virulence phenotype that remains to be determined (Sun et al. 2021; Han et al. 2024). Notably, these genes were flanked by mobile genetic elements, suggesting that recombination and transposition events may have mediated their integration into the chromosome.

To further investigate the genomic organization of the *iro* locus, the chromosomes of hypervirulent reference strains SGH10, related to CG23-I, and NTUH-K2044 were included in comparative analyses as they are widely recognized as reference genomes for the study of hypervirulent *K. pneumoniae* and provide a framework for assessing the conservation and evolutionary dynamics of virulence-associated *loci* (Lam et al. 2018a, b; Wu et al. 2009).

Genomic context analyses revealed that the glycosyltransferase-encoding gene *iroB* was located within a chromosomal region associated with ICEKp10, an integrative and conjugative element known to mobilize the yersiniabactin and colibactin *loci* (Lam et al. 2018c). A similar genomic organization was observed in the reference strain NTUH-K2044; however, in this isolate, additional genes from the *iroBCDN* cluster were present within the same chromosomal region and are indicated in gray in Fig. 2A. This pattern was also evident in the circular genome maps (Fig. 2C), which showed that only three isolates carried chromosomal copies of *iroB* and *iroD.* Notably, ITUAC436 was the only isolate in which the complete ICEKp10 region could be identified. PCR screening for the *clb* and *ybt* genes was performed, confirming the absence of ICEKp region in the other isolates (Supplementary Figure S1).

Figure 2B depicts the genomic context of the plasmid-associated *iro* *loci* in the sequenced isolates, alongside the reference virulence plasmid pLVPK (Chen et al. 2004). Moreover, the plasmid architecture observed in the Brazilian hvKp isolates was highly similar to that previously described for canonical virulence plasmids in hvKp. Additionally, two important hvKp biomarkers, *peg-344* and *rmpA*, were consistently identified within these plasmid regions, further supporting their role as virulence-associated plasmid backbones.

These findings, together with the plasmid reconstruction analysis (Fig. 2D), reaffirm the presence of hypervirulence-associated pKpVL-like plasmids among the sequenced isolates. Furthermore, the coexistence of chromosomal and plasmid-borne copies of *iroB* and *iroD* in isolates OBAC799, OBAC840, and ITUAC436 suggests an enhanced virulence potential. Although the phenotypic consequence of this genetic configuration remains to be experimentally validated, it may contribute to enhanced virulence potential in these isolates.

### Functional annotation of hypothetical proteins associated with hvKp isolates

Among the 53-protein clusters exclusively identified in hvKp isolates, four encoded hypothetical proteins (B382654, B382870, B382331 and B382547) previously highlighted by Spadar et al. (2022) as potentially virulence-associated markers through a genome-wide association study (GWAS) of hvKp isolates recovered from invasive liver infections. To investigate their possible biological functions and potential contribution to hypervirulence, these proteins were further characterized using the PROST protocol (Kilinc et al. 2022), combining homology-based and structural-based annotations.

Based on combined homology- and structure-based annotations, the following annotations were achieved: (i) protein B382654 = “putative carboxymuconolactone decarboxylase family protein”; (ii) protein B382870 = “putative DUF1471 domain-containing protein,”; (iii) protein B382331 = “putative DUF1471 domain-containing protein”; and (iv) protein B382547 = “putative DUF417 family protein”.

Previous studies in *K. pneumoniae* and *Escherichia coli* have already suggested an association of proteins containing the DUF1471 domain with biofilm formation and with responses to environmental stresses such as pH and temperature (Eletsky et al. 2014; Luo et al. 2017; Elistratova et al. 2022). Particularly, in *K. pneumoniae* it was suggested an inverse relationship between the expression of a DUF1471-containing protein and biofilm production. This aligns with other studies suggesting that strong biofilm formation is rarely correlated with the hypermucoviscous phenotype (Neumann et al. 2023). However, the relationship between the hypermucoviscous phenotype and biofilm development in hypervirulent hvKp strains remains a controversial matter. Mba et al. (2025), for instance, suggested a link between greater capsular thickness and biofilm development. Furthermore, Cubero et al. (2019) demonstrated in vitro, using an air-liquid interface system, that hvKp isolates of the K1 serotype carrying *magA* and *rmpA* produced more biofilm, despite lowered initial adhesion. Conversely, other authors suggest that hvKp isolates producing higher amounts of capsular polysaccharide (CPS) typically form less biofilm. Yao et al. (2025) suggested that *rmpA*, in addition to regulating CPS production, binds to the promoter region of the *mrk* locus, which encodes type 3 fimbriae essential for biofilm formation, and represses its expression. Teng et al. (2024) and Wang et al. (2023) showed that reduced CPS production and increased biofilm formation are inversely linked, occurring via promoter deletion of *rmpA* or deletion of *wcaJ*. In *K. pneumoniae*, Horng et al. (2021) described how deletion of the *orfX* gene, which encodes a DUF1471 domain-containing protein, increased biofilm production. Additionally, David et al. (2024) demonstrated increased expression of genes coding for DUF1471 domain-proteins following immune activation, reinforcing a potential mechanism in virulence. Consequently, we hypothesize that proteins containing the DUF1471 domain may contribute to the regulation of CPS production in hvKp isolates. In contrast, no clear associations have linked either the carboxymuconolactone decarboxylase family protein or DUF417 family protein domains to specific functions in Enterobacteriaceae to date.

**Fig. 3:**
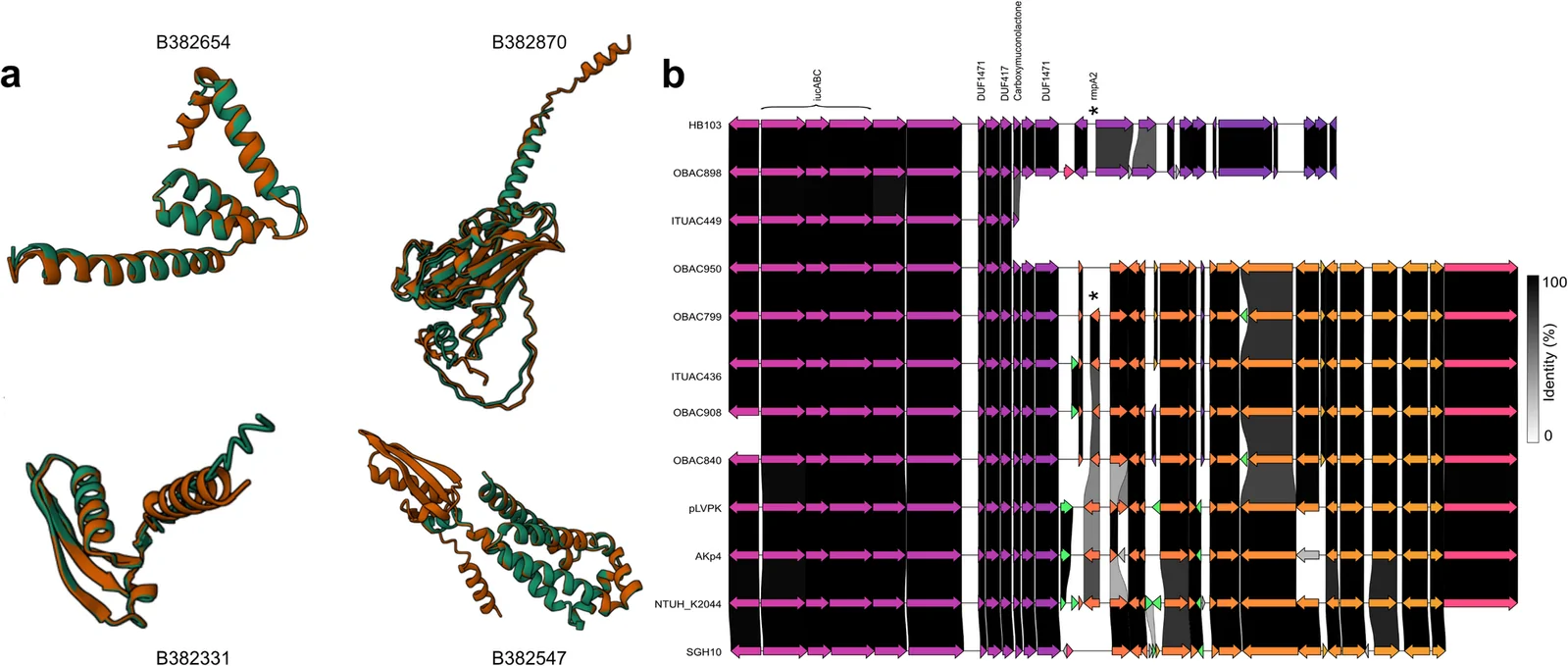
Annotations of hypothetical proteins exclusively-associated with hvKp isolates. (**A**) Structural superposition of hypothetical proteins encoded on plasmids from hypervirulent *K. pneumoniae* isolates associated with invasive liver infections. Alignments are shown for B382654 and a carboxymuconolactone decarboxylase family protein, B382870 and a DUF1471 domain-containing protein, B382331 and a DUF1471 domain-containing protein, B382547 and a DUF417 family protein. (**B**) Genomic context of hypothetical protein genes in hypervirulent *K. pneumoniae* isolates sequenced from Brazil. The asterisk (*) indicates the rmpA2 gene

In addition to functional annotation, structural overlap analyses were performed. As shown in Fig. 3A, the hypothetical proteins exhibited structural superposition across more than 90% of their length. The main structural differences were restricted to the terminal regions.

These hypothetical proteins are located downstream of the *iucABC* operon, which encodes the siderophore aerobactin, and upstream of the *rmpA2* gene (Fig. 3B). Both aerobactin and *RmpA2* are key biomarkers of the hypervirulent phenotype and are among the five markers used to identify hypervirulent *K. pneumoniae* isolates, achieving an accuracy of approximately 94% (Russo et al. 2024a).

Beyond its strong epidemiological association with hypervirulence, aerobactin has been shown to play a direct role in pathogenicity. Experimental studies demonstrated that deletion of the *iucA* gene results in a marked reduction in siderophore production and a substantial increase in the LD₅₀ in animal infection models, highlighting the critical contribution of aerobactin to virulence (Russo et al. 2024b).

### Implications for local surveillance

The identification of high-risk hvKp clones belonging to clonal groups CG23, CG65, CG86 and CG380 in Salvador expands the known geographic distribution of these lineages to northeastern Brazil. While these isolates remain largely drug-susceptible, as tested by the broth microdilution method using the VITEK 2 system, and six of them harbored IncHI1B(pNDM-MAR)-type and/or repB_KLEB replicons. This assignment refers to the plasmid replication module and does not indicate the presence of the resistance backbone with which these replicons have elsewhere been associated.

The hvKp-specific genomic characteristics identified in this study reinforce current knowledge regarding the genetic basis of hypervirulence and add to the still limited number of genomic descriptions of hvKp isolates from northeastern Brazil. These findings are particularly relevant given the limited number of reports describing hypervirulent *K. pneumoniae* isolates in Brazil, especially in this region (Morgado et al. 2022; Monteiro et al. 2025). Continued surveillance is essential to monitor the emergence and dissemination of these high-risk lineages and to detect potential convergence events at an early stage.

## Conclusions

This study provides a comparative genomic characterization of hvKp circulating in Salvador, Brazil, expanding the small number of hypervirulent lineages documented in the country. Eight isolates meeting the established genomic criteria for hvKp (*iucA*, *iroB*, *rmpA/A2* and *peg-344*) were characterized, they belonged to ST23, ST65 and ST86, were associated with K1 or K2 capsular types and the O1 antigen, and harbored few antimicrobial resistance genes. In addition, two less prevalent sequence types, ST3690 and ST5750, were identified among Brazilian hvKp isolates.

Four hypothetical proteins previously associated with invasive liver infections were annotated through combined sequence- and structure-based prediction. In addition, *iroB* and *iroD* were identified on both the chromosome and plasmids in three isolates, with the chromosomal copy located adjacent to an ICEKp10-associated region. Whether this genomic context reflects mobilization of the *iro* locus into the chromosome remains to be determined. Comparative genomic analyses further highlighted the open pangenome structure of *K. pneumoniae* and identified genes associated with genomic plasticity and horizontal gene transfer.

The isolates predominantly carried IncHI1B(pNDM-MAR)-like and repB_KLEB replicons, a plasmid background reported elsewhere to accommodate both virulence and resistance determinants. Collectively, these findings document the circulation of hvKp lineages in northeastern Brazil and may expand current knowledge of hvKp-associated genomic features and reinforce the need for integrated genomic surveillance to enable the early detection of MDR-hvKp emergence.

Limitations of this study: despite characterizing twenty-five genomes from Brazil, only eight of these *K. pneumoniae* isolates were hypervirulent, all recovered in a single city, which restricts the generalizability of our findings. Besides, all genomes were generated using short-read sequencing alone; although the assemblies met stringent quality thresholds, in the absence of hybrid short- and long-read assemblies, complete plasmid structures could not be reconstructed. Finally, no functional experiments were performed, so the mobility of the replicons described here remains to be demonstrated experimentally, as well as the contribution to the hypervirulence phenotype of the protein clusters enriched among hvKp isolates.

## Supplementary Information

Below is the link to the electronic supplementary material.

Supplementary Material 1

Supplementary Material 2

## Data Availability

The data generated during the current study are available in the European Nucleotide Archive (ENA) repository under PRJEB90215 project.

## References

[CR1] Abramson J, Adler J, Dunger J et al (2024) Accurate structure prediction of biomolecular interactions with AlphaFold 3. Nature 630:493–500. 10.1038/s41586-024-07487-w PMC11168924 38718835

[CR2] Arcari G, Carattoli A (2023) Global spread and evolutionary convergence of multidrug-resistant and hypervirulent *Klebsiella pneumoniae* high-risk clones. Pathog Glob Health 117:328–341. 10.1080/20477724.2022.2121362 PMC10177687 36089853

[CR3] Arredondo-Alonso S et al (2018) Mlplasmids: a user-friendly tool to predict plasmid- and chromosome-derived sequences for single species. Microb Genom 4:e000224. 10.1099/mgen.0.000224 PMC6321875 30383524

[CR4] Bortolaia V, Kaas RS, Ruppe E et al (2020) ResFinder 4.0 for predictions of phenotypes from genotypes. J Antimicrob Chemother 75:3491–3500. 10.1093/jac/dkaa345 PMC7662176 32780112

[CR5] Brisse S, Fevre C, Passet V et al (2009) Virulent clones of *Klebsiella pneumoniae*: identification and evolutionary scenario based on genomic and phenotypic characterization. PLoS One 4:e4982. 10.1371/journal.pone.0004982 PMC2656620 19319196

[CR6] Brown CL, Mullet J, Hindi F et al (2022) MobileOG-db: a manually curated database of protein families mediating the life cycle of bacterial mobile genetic elements. Appl Environ Microbiol 88:e00991-22. 10.1128/aem.00991-22 PMC9499024 36036594

[CR7] Cantalapiedra CP, Hernández-Plaza A, Letunic I et al (2021) eggNOG-mapper v2: Functional Annotation, Orthology Assignments, and Domain Prediction at the Metagenomic Scale. Mol Biol Evol 38:5825–5829. 10.1093/molbev/msab293 PMC8662613 34597405

[CR8] Chaudhari NM, Gupta VK, Dutta C (2016) BPGA- an ultra-fast pan-genome analysis pipeline. Sci Rep 6:24373. 10.1038/srep24373 PMC4829868 27071527

[CR9] Chen Y-T, Chang H-Y, Lai Y-C et al (2004) Sequencing and analysis of the large virulence plasmid pLVPK of Klebsiella pneumoniae CG43. Gene 337:189–198. 10.1016/j.gene.2004.05.008 15276215

[CR10] Chen T, Ying L, Xiong L et al (2024) Understanding carbapenem-resistant hypervirulent Klebsiella pneumoniae: Key virulence factors and evolutionary convergence. hLife 2:611–624. 10.1016/j.hlife.2024.06.005

[CR11] Cubero M et al (2019) Hypervirulent *Klebsiella pneumoniae* serotype K1 clinical isolates form robust biofilms at the air-liquid interface. PLoS One 14:e0222628. 10.1371/journal.pone.0222628 PMC6750583 31532800

[CR12] Cury J, Abby SS, Doppelt-Azeroual O et al (2020) Identifying conjugative plasmids and integrative conjugative elements with CONJscan. Methods Mol Biol 2075:265–283. 10.1007/978-1-4939-9877-7_19 31584169

[CR13] David C et al (2024) Intestinal carbapenem-resistant *Klebsiella pneumoniae* undergoes complex transcriptional reprogramming following immune activation. Gut Microbes 16:2340486. 10.1080/19490976.2024.2340486 PMC11057644 38659243

[CR14] Dong N, Yang X, Chan E-C et al (2022) *Klebsiella* species: taxonomy, hypervirulence and multidrug resistance. Ebiomedicine 79:103998. 10.1016/j.ebiom.2022.103998 PMC9010751 35405387

[CR15] Eletsky A, Michalska K, Houliston S et al (2014) Structural and functional characterization of DUF1471 domains of *Salmonella* proteins SrfN, YdgH/SssB, and YahO. PLoS One 9:e101787. 10.1371/journal.pone.0101787 PMC4092069 25010333

[CR16] Elistratova AA, Matrosova LE, Khilyas IV et al (2022) *Serratia marcescens* DUF1471-containing protein SrfN is needed for adaptation to acid and oxidative stresses. mSphere 7:e00212-22. 10.1128/msphere.00212-22 PMC9769812 36218346

[CR17] Gilchrist CLM, Booth TJ, Van Wersch B et al (2021) cblaster: a remote search tool for rapid identification and visualization of homologous gene clusters. Bioinformatics Adv 1:vbab016. 10.1093/bioadv/vbab016 PMC9710679 36700093

[CR18] Grant JR, Enns E, Marinier E et al (2023) Proksee: in-depth characterization and visualization of bacterial genomes. Nucleic Acids Res 51:W484–W492. 10.1093/nar/gkad326 PMC10320063 37140037

[CR19] Hallal Ferreira Raro O, Nordmann P, Dominguez Pino M et al (2023) Emergence of carbapenemase-producing hypervirulent *Klebsiella pneumoniae* in Switzerland. Antimicrob Agents Chemother. 10.1128/aac.01424-22 PMC10019205 36853006

[CR20] Han X, Zhou J, Yu L et al (2024) Genome sequencing unveils *blaKPC-2*-harboring plasmids as drivers of enhanced resistance and virulence in nosocomial *Klebsiella pneumoniae*. mSystems 9:e00924-23. 10.1128/msystems.00924-23 PMC10878039 38193706

[CR21] Hentzien M et al (2017) Seven hypervirulent ST380 Klebsiella pneumoniae septic localizations. Med Mal Infect 47:171–173. 10.1016/j.medmal.2016.10.002 27838216

[CR22] Horng Y-T et al (2022) A protein containing the DUF1471 domain regulates biofilm formation and capsule production in Klebsiella pneumoniae. J Microbiol Immunol Infect 55:1246–1254. 10.1016/j.jmii.2021.11.005 34924339

[CR23] Ito R, Mustapha MM, Tomich AD et al (2017) Widespread fosfomycin resistance in Gram-negative bacteria attributable to the chromosomal *fosA* gene. mBio 8:e00749-17. 10.1128/mbio.00749-17 PMC5574708 28851843

[CR24] Van Kempen M, Kim SS, Tumescheit C et al (2024) Fast and accurate protein structure search with Foldseek. Nat Biotechnol 42:243–246. 10.1038/s41587-023-01773-0 PMC10869269 37156916

[CR25] Kilinc M, Jia K, Jernigan RL (2022) Protein Language Model Performs Efficient Homology Detection. bioRxiv. 10.1101/2022.03.10.483778

[CR26] Lam MMC, Wyres KL, Duchêne S et al (2018a) Population genomics of hypervirulent *Klebsiella pneumoniae* clonal-group 23 reveals early emergence and rapid global dissemination. Nat Commun 9:2703. 10.1038/s41467-018-05114-7 PMC6045662 30006589

[CR27] Lam MMC, Wyres KL, Judd LM et al (2018b) Tracking key virulence loci encoding aerobactin and salmochelin siderophore synthesis in *Klebsiella pneumoniae*. Genome Med 10:77. 10.1186/s13073-018-0587-5 PMC6205773 30371343

[CR28] Lam MMC, Wyres KL, Wick RR et al (2018c) Genetic diversity, mobilisation and spread of the yersiniabactin-encoding mobile element ICEKp in Klebsiella pneumoniae populations. Microb Genom 4:e000196. 10.1099/mgen.0.000196 PMC6202445 29985125

[CR29] Lee C-R, Lee JH, Park KS et al (2017) Antimicrobial Resistance of Hypervirulent Klebsiella pneumoniae: Epidemiology, Hypervirulence-Associated Determinants, and Resistance Mechanisms. Front Cell Infect Microbiol 7:483. 10.3389/fcimb.2017.00483 PMC5702448 29209595

[CR30] Li Z, Jaroszewski L, Iyer M et al (2020) FATCAT 2.0: towards a better understanding of the structural diversity of proteins. Nucleic Acids Res 48:W60–W64. 10.1093/nar/gkaa443 PMC7319568 32469061

[CR31] Li Y, Kumar S, Zhang L et al (2023) Characteristics of antibiotic resistance mechanisms and genes of *Klebsiella pneumoniae*. Open Med 18:20230707. 10.1515/med-2023-0707 PMC10183727 37197355

[CR32] Li X, Chen S, Lu Y et al (2025) Molecular epidemiology and genetic dynamics of carbapenem-resistant hypervirulent *Klebsiella pneumoniae* in China. Front Cell Infect Microbiol 15:1529929. 10.3389/fcimb.2025.1529929 PMC11868059 40028179

[CR33] Linkevicius M, Alm E, Roer L et al (2025) Cross-border spread of a mosaic resistance (OXA-48) and virulence (aerobactin) plasmid in *Klebsiella pneumoniae*: a European Antimicrobial Resistance Genes Surveillance Network investigation, Europe, February 2019 to October 2024. Euro Surveill 30:2500439. 10.2807/1560-7917.ES.2025.30.27.2500439 PMC12262110 40642770

[CR34] Loiodice M et al (2025) Molecular epidemiology of Klebsiella pneumoniae causing bloodstream infections in a district hospital in Northern Portugal (2016–2018): clonal diversity and detection of hypervirulent strains. Eur J Clin Microbiol Infect Dis 44:3087–3095. 10.1007/s10096-025-05260-z 41023356

[CR35] Luo M, Yang S, Li X et al (2017) The KP1_4563 gene is regulated by the cAMP receptor protein and controls type 3 fimbrial function in *Klebsiella pneumoniae* NTUH-K2044. PLoS ONE 12:e0180666. 10.1371/journal.pone.0180666 PMC5521740 28732013

[CR36] Maguire M, DeLappe N, Clarke C et al (2025) Genomic and phylogenetic analysis of hypervirulent Klebsiella pneumoniae ST23 in Ireland. Microb Genom 11:001373. 10.1099/mgen.0.001373 PMC12452180 40106330

[CR37] Mba IE et al (2025) New insights and perspectives on the virulence of hypervirulent Klebsiella pneumoniae. Folia Microbiol 70:517–533. 10.1007/s12223-025-01261-9 40198504

[CR38] Medina VA et al (2025) Epidemiological genomics of *Klebsiella pneumoniae* isolates from hospitals across Colombia. npj Antimicrob Resist 3:64. 10.1038/s44259-025-00127-x PMC12280007 40691499

[CR39] Monteiro ADSS, Cordeiro SM, Reis JN (2024) Virulence Factors in Klebsiella pneumoniae: A Literature Review. Indian J Microbiol 64:389–401. 10.1007/s12088-024-01247-0 PMC11246375 39011017

[CR40] Monteiro ADSS, Silva MDO, Galvão VS et al (2025) High proportions of multidrug-resistant *Klebsiella pneumoniae* isolates in community-acquired infections, Brazil. Sci Rep 15:9698. 10.1038/s41598-025-92549-w PMC11926192 40113819

[CR41] Morgado S, Fonseca E, Vicente AC (2022) Genomics of *Klebsiella pneumoniae* species complex reveals the circulation of high-risk multidrug-resistant pandemic clones in human, animal, and environmental sources. Microorganisms 10:2281. 10.3390/microorganisms10112281 PMC9697336 36422351

[CR42] Navon-Venezia S, Kondratyeva K, Carattoli A (2017) Klebsiella pneumoniae: a major worldwide source and shuttle for antibiotic resistance. FEMS Microbiol Rev 41:252–275. 10.1093/femsre/fux013 28521338

[CR43] Néron B, Denise R, Coluzzi C et al (2023) MacSyFinder v2: improved modelling and search engine to identify molecular systems in genomes. Peer Community J 3:e28. 10.24072/pcjournal.250

[CR44] Neumann B et al (2023) Detection and characterization of putative hypervirulent *Klebsiella pneumoniae* isolates in microbiological diagnostics. Sci Rep 13:19025. 10.1038/s41598-023-46221-w PMC10624845 37923898

[CR45] Olson RD, Assaf R, Brettin T et al (2023) Introducing the Bacterial and Viral Bioinformatics Resource Center (BV-BRC): a resource combining PATRIC, IRD and ViPR. Nucleic Acids Res 51:D678–D689. 10.1093/nar/gkac1003 PMC9825582 36350631

[CR46] Rodríguez-Medina N et al (2024) Comprehensive study reveals phenotypic heterogeneity in *Klebsiella pneumoniae* species complex isolates. Sci Rep 14:5876. 10.1038/s41598-024-55546-z PMC10928225 38467675

[CR47] Russo TA, Marr CM (2019) Hypervirulent *Klebsiella pneumoniae*. Clin Microbiol Rev 32:e00001-19. 10.1128/CMR.00001-19 PMC6589860 31092506

[CR48] Russo TA, Alvarado CL, Davies CJ et al (2024a) Differentiation of hypervirulent and classical *Klebsiella pneumoniae* with acquired drug resistance. mBio 15:e02867-23. 10.1128/mbio.02867-23 PMC10865842 38231533

[CR49] Russo TA, Carlino-MacDonald U, Drayer ZJ et al (2024b) Deciphering the relative importance of genetic elements in hypervirulent Klebsiella pneumoniae to guide countermeasure development. eBioMedicine 107:105302. 10.1016/j.ebiom.2024.105302 PMC11388194 39178743

[CR50] Sehnal D, Bittrich S, Deshpande M et al (2021) Mol* Viewer: modern web app for 3D visualization and analysis of large biomolecular structures. Nucleic Acids Res 49:W431–W437. 10.1093/nar/gkab314 PMC8262734 33956157

[CR51] Sherry NL, Horan KA, Ballard SA et al (2023) An ISO-certified genomics workflow for identification and surveillance of antimicrobial resistance. Nat Commun 14:60. 10.1038/s41467-022-35713-4 PMC9813266 36599823

[CR52] Spadar A, Perdigão J, Campino S, Clark TG (2022) Genomic analysis of hypervirulent *Klebsiella pneumoniae* reveals potential genetic markers for differentiation from classical strains. Sci Rep 12:13671. 10.1038/s41598-022-17995-2 PMC9372168 35953553

[CR53] Spadar A, Perdigão J, Campino S, Clark TG (2023) Large-scale genomic analysis of global *Klebsiella pneumoniae* plasmids reveals multiple simultaneous clusters of carbapenem-resistant hypervirulent strains. Genome Med 15:3. 10.1186/s13073-023-01153-y PMC9850321 36658655

[CR54] Stanton TD, Wyres KL (2024) What defines hypervirulent *Klebsiella pneumoniae*? eBioMedicine 108:105331. 10.1016/j.ebiom.2024.105331 PMC11414540 39260040

[CR55] Sun L, Li H, Wang Q et al (2021) Increased gene expression and copy number of mutated blaKPC lead to high-level ceftazidime/avibactam resistance in Klebsiella pneumoniae. BMC Microbiol 21:230. 10.1186/s12866-021-02293-0 PMC8375111 34412588

[CR56] Sun J, Lu F, Luo Y et al (2023) OrthoVenn3: an integrated platform for exploring and visualizing orthologous data across genomes. Nucleic Acids Res 51:W397–W403. 10.1093/nar/gkad313 PMC10320085 37114999

[CR57] Teng G et al (2024) Adaptive attenuation of virulence in hypervirulent carbapenem-resistant *Klebsiella pneumoniae*. mSystems 9:e01363-23. 10.1128/msystems.01363-23 PMC11237801 38752758

[CR58] Wang W et al (2023) Different regulatory mechanisms of the capsule in hypervirulent *Klebsiella pneumonia*: “direct” wcaJ variation vs. “indirect” rmpA regulation. Front Cell Infect Microbiol 13:1108818. 10.3389/fcimb.2023.1108818 PMC10168181 37180440

[CR59] Wu K-M, Li L-H, Yan J-J et al (2009) Genome Sequencing and Comparative Analysis of Klebsiella pneumoniae NTUH-K2044, a Strain Causing Liver Abscess and Meningitis. J Bacteriol 191:4492–4501. 10.1128/JB.00315-09 PMC2704730 19447910

[CR60] Wyres KL, Wick RR, Gorrie C et al (2016) Identification of Klebsiella capsule synthesis loci from whole genome data. Microb Genom 2:e000102. 10.1099/mgen.0.000102 PMC5359410 28348840

[CR61] Wyres KL, Lam MMC, Holt KE (2020) Population genomics of Klebsiella pneumoniae. Nat Rev Microbiol 18:344–359. 10.1038/s41579-019-0315-1 32055025

[CR62] Yao S et al (2025) RmpA drives metabolic reprogramming to modulate the phenotypic switch between hypermucoviscosity and biofilm formation in hypervirulent Klebsiella pneumoniae. hLife 3:504–516. 10.1016/j.hlife.2025.06.005

[CR63] Zhang F, Li L, Zhao Y et al (2024) Molecular characterization of hybrid virulence plasmids in ST11-KL64 KPC-2-producing multidrug-resistant hypervirulent *Klebsiella pneumoniae* from China. Front Microbiol 15:1353849. 10.3389/fmicb.2024.1353849 PMC10972857 38550871

