# Supplementary material for: Comparative genomics of hypervirulent Klebsiella pneumoniae isolates from Brazil
Source: Arch Microbiol. 2026 Oct 12;209(1):17. doi: 10.1007/s00203-026-05262-1 (PMC13658379; doi:10.1007/s00203-026-05262-1)
Supplement: Supplementary file 1 [file 203_2026_5262_MOESM2_ESM.docx]

**Supplementary Materials**

**Comparative genomics of hypervirulent *Klebsiella pneumoniae* isolates from Brazil**

Felipe G. Sacoda^a^, João V. O. Cruz^a^, Itana A. Santos^a^, Adriano S. S. Monteiro^b,c^, Amanda O. S. Melo^c^, Danilo J. P. G. Rocha^d^, Emilia M. M. A. Belitardo^d^, Flavia F. Aburjaile^e^, Siomar C. Soares^f^, Mateus M. Costa^g^, Aristóteles Góes-Neto^h^, Rommel T. J. Ramos^i^, Bertram Brenig^j^, Vasco Azevedo^h^, Joice N. Reis^b,c^, and Luis G. C. Pacheco^a,b,d^ *

^a^ Post-Graduate Program in Biotechnology, Federal University of Bahia, Salvador-BA, Brazil.

^b^ Gonçalo Moniz Institute, Oswaldo Cruz Foundation (FIOCRUZ), Salvador-BA, Brazil.

^c^ Faculty of Pharmacy, Federal University of Bahia, Salvador-BA, Brazil.

^d^ Institute of Health Sciences, Federal University of Bahia, Salvador-BA, Brazil.

^e^ Department of Preventive Veterinary Medicine, Federal University of Minas Gerais, Belo Horizonte-MG, Brazil.

^f^ Institute of Biological and Natural Sciences, Federal University of Triângulo Mineiro, Uberaba-MG, Brazil.

^g^ Federal University of the São Francisco Valley, Petrolina-PE, Brazil.

^h^ Institute of Biological Sciences, Federal University of Minas Gerais, Belo Horizonte-MG, Brazil.

^i^ Federal University of Pará, Belém-PA, Brazil.

^j^ Institute of Veterinary Medicine, University of Göttingen, 37073 Göttingen, Germany.

Correspondence: L. Pacheco. Gonçalo Moniz Institute, Oswaldo Cruz Foundation (IGM-Fiocruz), Salvador-BA, Brazil. E-mail: luis.pacheco@fiocruz.br

Supplementary table 1 - Assembly and annotation statistics for 25 sequenced *Klebsiella pneumoniae* isolates from Salvador, Bahia, Brazil

| Samples Name | Number of contigs | G+C% Content | L50 | Genome size | N50 | Sequencing coverage | Completeness | tRNA | Number of coding sequences |
| --- | --- | --- | --- | --- | --- | --- | --- | --- | --- |
| OBAC799 | 38 | 57.23 | 4 | 5,511,551 | 440,925 | 168,961 | 100 | 75 | 5,389 |
| OBAC840 | 46 | 57.25 | 5 | 5,531,322 | 365,715 | 188,137 | 100 | 77 | 5,436 |
| OBAC898 | 33 | 57.24 | 3 | 5,388,700 | 499,608 | 162,555 | 100 | 77 | 5,265 |
| OBAC908 | 48 | 57.07 | 7 | 5,420,545 | 303,481 | 190,182 | 100 | 76 | 5,329 |
| OBAC950 | 45 | 57.29 | 6 | 5,374,056 | 335,825 | 182,077 | 100 | 76 | 5,321 |
| HB103 | 45 | 57.29 | 6 | 5,374,056 | 335,825 | 154,227 | 100 | 72 | 5,321 |
| ITUAC436 | 45 | 57.29 | 6 | 5,374,056 | 335,825 | 101,203 | 100 | 75 | 5,321 |
| ITUAC449 | 70 | 57.26 | 12 | 5,304,412 | 168,872 | 167,272 | 100 | 75 | 5,209 |
| HB083 | 92 | 57.05 | 8 | 5,838,199 | 239,778 | 166,102 | 100 | 79 | 5,841 |
| HSR187 | 149 | 57.28 | 9 | 5,476,554 | 209,249 | 150,713 | 100 | 76 | 5,487 |
| HSR496 | 62 | 57.40 | 5 | 5,364,213 | 427,2 | 161,647 | 100 | 72 | 5,259 |
| HSR510 | 151 | 56.67 | 11 | 5,755,622 | 173,338 | 96,9 | 100 | 77 | 5,797 |
| HSR544 | 140 | 57.34 | 8 | 5,756,333 | 279,949 | 155,884 | 100 | 81 | 5,821 |
| HB044 | 77 | 57.18 | 6 | 5,562,200 | 328,771 | 151,743 | 100 | 78 | 5,539 |
| HB068 | 77 | 57.18 | 6 | 5,562,181 | 328,771 | 175,828 | 100 | 78 | 5,541 |
| HB070 | 92 | 57.09 | 6 | 5,658,344 | 328,771 | 194,402 | 100 | 78 | 5,672 |
| HSR410 | 94 | 57.18 | 6 | 5,564,550 | 328,771 | 157,864 | 100 | 78 | 5,542 |
| ICOM45 | 92 | 57.05 | 8 | 5,840,148 | 239,832 | 174,475 | 100 | 77 | 5,843 |
| ICOM68 | 113 | 56.77 | 7 | 5,835,214 | 252,903 | 135,26 | 100 | 74 | 5,863 |
| ICOM128 | 94 | 57.04 | 6 | 5,700,628 | 349,173 | 153,764 | 100 | 78 | 5,726 |
| ICOM203 | 80 | 57.17 | 6 | 5,561,909 | 328,771 | 147,354 | 100 | 78 | 5,537 |
| ICOM329 | 123 | 56.56 | 9 | 5,950,831 | 229,274 | 153,566 | 100 | 78 | 6,045 |
| ICOM343 | 103 | 57.00 | 9 | 5,898,443 | 212,807 | 171,909 | 100 | 79 | 5,914 |
| ICOM692 | 115 | 56.90 | 10 | 5,772,897 | 182,265 | 146,341 | 99.9 | 77 | 5,824 |
| ICOM779 | 124 | 57.06 | 9 | 5,778,071 | 219,954 | 183,904 | 100 | 77 | 5,823 |

Supplementary table 2 - Annotation and GenBank ID of proteins present in clusters of hypervirulent *K. pneumoniae* isolates.

|  | Protein_ID | Annotation | Location |
| --- | --- | --- | --- |
| 1 | WLX18656.1 | small membrane protein | Chromosome |
| 2 | WLX18679.1 | KPN_01571 family protein | Chromosome |
| 3 | WLX18680.1 | carboxypeptidase M32 | Chromosome |
| 4 | WLX18682.1 | serine protease | Chromosome |
| 5 | WLX18683.1 | ArsR family transcriptional regulator | Chromosome |
| 6 | WLX18684.1 | multidrug efflux SMR transporter subunit KpnF | Chromosome |
| 7 | WLX18685.1 | multidrug efflux SMR transporter subunit KpnE | Chromosome |
| 8 | WLX18686.1 | AI-2E family transporter | Chromosome |
| 9 | WLX20320.1 | polysaccharide pyruvyl transferase family | Chromosome |
| 10 | WLX20321.1 | glycosyltransferase | Chromosome |
| 11 | WLX20322.1 | PAAR domain-containing protein | Chromosome |
| 12 | WLX20323.1 | hypothetical protein | Chromosome |
| 13 | WLX22602.1 | glycosyltransferase | Chromosome |
| 14 | WLX22603.1 | alpha/beta hydrolase-fold protein | Chromosome |
| 15 | WLX22898.1 | histone-like nucleoid-structuring protein H-NS | Chromosome |
| 16 | WLX22941.1 | hypothetical protein | Chromosome |
| 17 | WLX23215.1 | CcdB family protein | Chromosome |
| 18 | WLX23576.1 | hypothetical protein | Plasmid |
| 19 | WLX23577.1 | DMT family inner membrane transporter PEG344 | Plasmid |
| 20 | WLX23578.1 | mucoid phenotype regulator RmpA | Plasmid |
| 21 | WLX23579.1 | class I SAM-dependent methyltransferase | Plasmid |
| 22 | WLX23587.1 | GTP-binding protein | Plasmid |
| 23 | WLX23588.1 | transcriptional repressor | Plasmid |
| 24 | WLX23589.1 | c-type lysozyme inhibitor | Plasmid |
| 25 | WLX23593.1 | NIS family aerobactin synthetase IucA | Plasmid |
| 26 | WLX23594.1 | N(6)-hydroxylysine O-acetyltransferase IucB | Plasmid |
| 27 | WLX23595.1 | NIS family aerobactin synthetase IucC | Plasmid |
| 28 | WLX23597.1 | DUF1471 domain-containing protein | Plasmid |
| 29 | WLX23598.1 | TetR/AcrR family transcriptional regulator | Plasmid |
| 30 | WLX23599.1 | DUF417 family protein | Plasmid |
| 31 | WLX23600.1 | carboxymuconolactone decarboxylase family protein | Plasmid |
| 32 | WLX23601.1 | DM13 domain-containing protein | Plasmid |
| 33 | WLX23602.1 | DUF1471 domain-containing protein | Plasmid |
| 34 | WLX23616.1 | Tn3 family transposase | Plasmid |
| 35 | WLX23617.1 | alpha/beta hydrolase | Plasmid |
| 36 | WLX23640.1 | DUF2251 domain-containing protein | Plasmid |
| 37 | WLX23641.1 | DUF1778 domain-containing protein | Plasmid |
| 38 | WLX23667.1 | ParB/RepB/Spo0J family plasmid partition protein | Plasmid |
| 39 | WLX23668.1 | plasmid-partitioning protein SopA | Plasmid |
| 40 | WLX23669.1 | RepB family plasmid replication initiator protein | Plasmid |
| 41 | WLX23670.1 | plasmid transfer protein | Plasmid |
| 42 | WLX23717.1 | DUF1173 family protein | Plasmid |
| 43 | WLX23733.1 | zinc metallochaperone GTPase ZigA | Plasmid |
| 44 | WLX23739.1 | RNA polymerase-binding protein DksA | Plasmid |
| 45 | WLX23760.1 | DUF2384 domain-containing protein | Plasmid |
| 46 | WLX23762.1 | TonB-dependent Fe(3+) dicitrate receptor FecA | Plasmid |
| 47 | WLX23763.1 | ferric citrate uptake sigma factor FecI | Plasmid |
| 48 | WLX23767.1 | salmochelin/enterobactin export ABC transporter IroC | Plasmid |
| 49 | WLX23768.1 | IS3 family transposase | Plasmid |
| 50 | WLX23772.1 | recombinase family protein | Plasmid |
| 51 | WLX23774.1 | mucoid phenotype regulator RmpC | Plasmid |
| 52 | WLX23775.1 | mucoid phenotype synthesis protein RmpD | Plasmid |
| 53 | WLX23777.1 | MFS transporter | Plasmid |

Supplementary Figure 1 - Agarose gel electrophoresis of the PCR products obtained for the *ybt* (yersiniabactin locus) and *clb* (colibactin locus) targets in the eight hvKp isolates from Salvador, Brazil. Reactions were performed with the GoTaq® G2 Hot Start Polymerase kit.

**Supplementary File 2- Excel file**
ESM1. Mobile genetic element-associated proteins identified in the eight hypervirulent *Klebsiella pneumoniae* isolates by mobileOG-db. Each worksheet corresponds to one isolate and lists all retained hits with their mobileOG-db identifiers. Functional categories follow the mobileOG-db classification scheme: integration/excision, replication/recombination/repair, stability/transfer/defense, transfer and phage.
